# The MacBrain Resource Center (MBRC) rhesus macaque embryonic brain histology datasets

**DOI:** 10.1111/joa.70161

**Published:** 2026-04-21

**Authors:** Valeria Mendoza‐Silva, Emma Burke, Lucy Greene, Mikhail Rodov, Janja Kopić, Phil Barello, Yury M. Morozov, Keiko Moriya‐Ito, Chiaki Ohtaka‐Maruyama, Zeljka Krsnik, Pasko Rakic, Alvaro Duque

**Affiliations:** ^1^ MacBrain Resource Center (MBRC)—Department of Neuroscience Yale University School of Medicine New Haven Connecticut USA; ^2^ Croatian Institute for Brain Research University of Zagreb School of Medicine Zagreb Croatia; ^3^ Tokyo Metropolitan Institute of Medical Sciences Tokyo Japan

**Keywords:** cerebral cortex, databases, factual, embryo, mammalian, immunohistochemistry, *Macaca mulatta*, microscopy, electron, neurogenesis, tissue banks

## Abstract

Datasets for the study of developing Non‐Human Primate (NHP) brain are scarce. Some of the reasons are related to the financial and technical obstacles that make the production of these datasets logistically challenging. NHP embryos of different and verified developmental ages require timed pregnancies and the maintenance of expensive and highly regulated breeding colonies. Here, we document our efforts to provide digital datasets of rhesus macaque (*Macaca mulatta*) embryonic brain histological series of sections that can be used and reused for research. These efforts fall well within the primary mission of the MacBrain Resource Center (MBRC, https://medicine.yale.edu/neuroscience/macbrain/mission/), which targets a reduction in the need to sacrifice animals, improved efficiency in the use of resources, the use of male (M) and female (F) specimens to conduct developmental research, and increase archival and sharing of materials for the advancement of neuroscience. First, we describe the already existent tritiated thymidine (^3^H‐TdR) cases in MBRC Collection 1 and the electron microscopy (EM) blocks of Collection 5, which together contain >100 specimens of different developmental ages. We also report methodological details on, as of July 2025, *n* = 24 histo‐ and immunohistochemically processed embryonic brains that enrich Collection 6 with thousands of coronal sections stained for *n* = 38 different cellular and fiber markers. These datasets provide NHP brain histo‐ and immunohistology for comparison with other species and hence help close a gap in the availability of suitable materials for comparative evolution and neurodevelopmental studies. Through examples, we illustrate how different materials are currently being used in de novo research. Finally, we advocate the absolute need to continue using NHPs in the study of neurodevelopment because of the unparalleled molecular, genetic, and anatomical similarities between these animal models and humans.

## Graphical abstract

We introduce the MacBrain Resource Center (MBRC) Collections of rhesus macaque embryonic brain tissue. Here, we illustrate histo‐ and immunohistology from our dynamically growing Collection 6, which currently contains >10,000 zoomable and downloadable images from *n* = 14 male and female embryos from early to late in gestation. (A–C) Low magnification panoramic views showing (A) NeuN, (B) AChE, (C) Nissl, (D) TH in a female embryo age 93 days (E93). Coronal sections are from a rostro‐caudal level close to the crossing of the anterior commissure. The scale bar applies to all panels. AC, anterior commissure; Cn, caudate nucleus; GP, globus pallidus; ic, internal capsule; Put, putamen. The sections are from B175 publicly accessible at https://macbraingallery.yale.edu/collection6/.

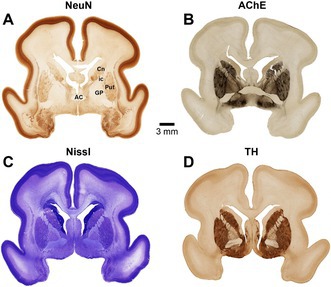

## 1 INTRODUCTION

The study of the cellular and molecular mechanisms involved in the regulation of cell numbers, migration, disposition, and circuit assembly of the human cerebral cortex is at the very core of the symposium summarized in the present special issue. However, the study of human cerebral cortex development would be seriously limited if it were not for the use of a host of animal models that permit experimental manipulations (Gilardi & Kalebic, 2021; Vanderhaeghen & Polleux, 2023). Among these are many mouse lines specifically designed for the study of the effects of genetic changes, in vivo and in vitro electrophysiology, optogenetics, genomics, proteomics, and many other research venues (Boyden et al., 2005; Capecchi, 2005; Daigle et al., 2018; Luhmann & Fukuda, 2020; Yue et al., 2014). Of course, depending on the question at hand, rodents may provide advantages in comparison to primates or other species. Most strains of the common laboratory rat (*Rattus norvegicus domesticus*) or mouse (*Mus musculus domesticus*) have gestational periods of just 19–21 days (Elizalde‐Bielsa et al., 2024; Pritchett‐Corning et al., 2013), and in many cases, more than a dozen animals are born at a time. Characteristics that can help speed up studies of brain development. In the laboratory setting, life expectancy is ~2 years for both species (Arellano et al., 2024; Kawakami et al., 2022; Sengupta, 2013). The size of the rat and mouse brains, compared to that of the rhesus macaque (*Macaca mulatta*), is much smaller, decreasing laboratory consumables and processing time, ultimately decreasing expenses (Herculano‐Houzel et al., 2007). Unfortunately, these advantages constitute shortcomings when investigating some important features of human cortical development, such as prolonged neurogenesis, gyrification, expanded subplate compartments, extended windows of neuronal migration and circuit maturation, all of which are absent or highly compressed in rodents (Nano & Bhaduri, 2022). These differences limit the direct extrapolation of rodent developmental timelines and cytoarchitectonic patterns to the human brain (Azkona & Sanchez‐Pernaute, 2022; Kennedy & Dehay, 2020). In addition, directly working with human tissue presents unique ethical and legal issues regarding abortions, tissue donation, and collection. Also, human embryonic and fetal datasets are limited by the scarcity of high‐quality specimens because of factors such as variability in postmortem interval (PMI) and tissue handling that can further compromise tissue preservation and integrity (Dawood et al., 2023; Nagy et al., 2015; Olney et al., 2025). Parameters that, in the laboratory setting, can be controlled.

Particularly useful for developmental, aging, and anatomical comparisons to human brains are brains from non‐human primates (NHPs) (Bakken et al., 2021; Duque et al., 2016; Qiao et al., 2023; Rakic, 2009). Among these, rhesus macaques and common marmosets (*Callithrix jacchus*) have developmental trajectories and general biological characteristics with both divergent and overlapping parameters, which make them complementary and very useful for comparative studies (Duque, 2023a, 2023b; Rakic, 2009). Among the commonalities, both have relatively long gestational periods, with an average 165‐day gestation in the rhesus and 152 days in the marmoset that provide an extended temporal window for experimentation and observation; both have rich social lives with abundant vocalization; and, by virtue of being primates, they share with humans a large percentage of their genomic and proteomic makeup (Coe & Lubach, 2021; Duque, 2023a, 2023b; Gibbs et al., 2007; Miller et al., 2016). Rhesus macaques have relatively large (~80–100 g) brains, while marmosets have relatively small (~7–8 g) brains (Herculano‐Houzel et al., 2007). Fertility viability in the rhesus starts between 3 and 4 years, while in marmosets, sexual maturity occurs earlier (~1.5–2 years) (Stephens & Wallen, 2013). Rhesus monkeys produce 1 offspring per pregnancy, and marmosets produce 2–4. In the laboratory setting, the lifespan in rhesus can be as long as ~30 years, and ~10 years in the case of marmosets (Colman et al., 2014; Tardif et al., 2011). Importantly, both are models of general interest in biomedical investigations and excel in their utility in neuroscience and brain developmental research because of their brain regionalization and cytoarchitectonic characteristics. However, between the rhesus macaque and marmoset, only the rhesus has a gyrencephalic brain.

Here, we summarize the current status of our efforts to provide a rich repertoire of rhesus macaque brain coronal sections processed for different histo‐ and immunohistological stains at different embryonic ages. We also provide examples of rhesus macaque developmental age in relationship to development in humans and offer examples of ongoing research using our unique materials. The complementary strengths of rhesus macaques and marmosets in the study of cortical development are discussed in the general venue to help advance our understanding of human normal and abnormal cortical development.

Our long‐term goals are severalfold. (1) To obtain sufficient data to establish a normative standard for developmental NHP studies. (2) To provide high‐resolution cytoarchitectonic data against which imaging obtained by magnetic resonance (MRI), ultrasound, and other techniques can be registered. (3) To ensure data sharing and reusability to decrease the number of animals needed to be sacrificed in future studies. (4) To obtain data from both M and F specimens so that sex as a biological variable can truly be evaluated during brain development.

We consider the establishment of standards essential for our understanding of normal developmental changes, which is in turn necessary to distinguish what changes can be considered pathological (Duque, 2023a, 2023b; Duque et al., 2025). Similarly, understanding sexual dimorphism as early in development as possible will pave the way to a better understanding of differences between M and F developmental vulnerability periods, which, when compromised, may result in clinical consequences much later in life. Finally, we encourage and, through the MBRC, promote the sharing of vast amounts of histological data to foster and facilitate NHP studies of higher scientific rigor that guarantee experimental repeatability in neurodevelopmental research.

## 2 MATERIALS AND METHODS

### 2.1 Animals

Rhesus macaque embryo specimens.

As reported in our companion paper in this issue (Mendoza‐Silva et al., 2026), many animals used in the production of Collection 1 (^3^H‐TdR) and Collection 5 (electron microscopy [EM] blocks) date from the late 1960s and early 1970s, while Dr. Pasko Rakic was still at Harvard. However, all the embryos processed for immunohistochemistry (Collection 6) have been produced and processed at Yale University. In all cases, animal studies were, and continue to be, conducted in accordance with all federal and state regulations and are reviewed and approved by the Institutional Animal Care and Use Committee (IACUC).

### 2.2 Histo‐ and immunohistochemistry

**FIGURE 1:**
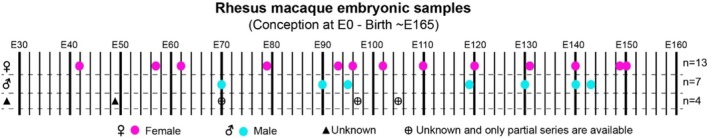
Rhesus macaque embryonic samples processed as of July 2025 (*n* = 24) for different cellular, extracellular, and fiber stains. The three specimens in which only partial series are available were from experiments in which only rostral and caudal blocks were left from unrelated experiments, and hence they do not contain complete rostro‐caudal series; the available coronal sections of those samples were cut at 30 μm, while everything else was cut at 50 μm. In addition, they are the only ones in which the pregnant NHP was injected with the thymidine analog bromodeoxyuridine (BrdU) prior to embryo extraction by C‐section. Consequently, they are the only ones, at this time, in which BrdU staining is also available.

To afford the most comprehensive and complete collection of NHP brain sections processed for histo‐ and immunohistochemical comparison to human, the MBRC is currently systematically processing a series of coronal sections from M and F rhesus macaque embryos of different ages (E). Our target is a minimum of 3 M and 3 F per age point from approximately E30 to E150 spaced, on average, 5–10 days apart. Consecutive sections are processed for a battery of different stains at different sampling rates. Since markers start to be detectable at different ages, specific stains are selected for targeting the developmental profile of explicit cellular and fiber populations of interest according to developmental age. The number of series per brain and the number of sections per series are balanced in an effort to obtain as many series and stains as possible per sample. Naturally, age and consequently brain size are major factors limiting the number of sections and the corresponding number of different series of the same stain that can be obtained from a sample. Figure 1 shows the age and sex distribution of the current samples (*n* = 24 total; *n* = 22 already processed and *n* = 2 in process). Table 1 (abbreviations) lists the *n* = 38 markers used so far.

**TABLE 1:** Immunohistology abbreviations and primary antibody details for neuronal, glial, and fiber markers.

Abbreviation	Definition	Host	Description	Catalog	RRID	Source	Dilution
5‐HT	Serotonin	Rb	Poly	20080	AB_572263	ImmS	1:10 K
BIII tubulin	Beta III tubulin	Ms, CLTuJ‐1	Mono	MAB1195	AB_357520	RDS	1:5 K
BrdU	Bromodeoxyuridine	Rt	Mono	AB6326	AB_305426	Abcam	1:2000
CB	Calbindin	Ms	Mono	C9848	AB_476894	MPS	1:1000
CCK	Cholecystokinin	Rb	Poly	MBS555736	NA	MBS	1:4000
ChAT	Choline acetyltransferase	Ms, CL3173	Mono	NBP2‐46620	AB_2922998	NVS	1:500
CR	Calretinin	Rb	Poly	CR7697	AB_2619710	Swant	1:10 K
DCX	Doublecortin	Rb	Poly	4604	AB_561007	CST	1:5 K
Fibronectin	Fibronectin	Rb	Poly	F3648	AB_476976	Sigma	1:1000
GFAP	Glial fibrillary acidic protein	Rb	Poly	Z0334	AB_10013382	DAKO	1:5 K
Iba1	Ionized Ca^2+^‐binding adaptor	Rb	Poly	019‐19741	AB_839504	Wako	1:8 K
Ki67	Ki67 antigen	Rb	Poly	AB15580	AB_443209	Abcam	1:5 K
MBP	Myelin basic protein	Rt, CL12	Mono	MAB386	AB_94975	MPS	1:200
NCAN	Neurocan	Rb	Poly	HPA036814	AB_10673666	Sigma	1:500
NeuN	Neuronal nuclear protein OR Fox3	Ms	Mono	MAB377	AB_2298772	MPS	1:3000
NOS	Nitric oxide synthase	Rb	Poly	61‐7000	AB_2313734	TF	1:1000
NPY	Neuropeptide Tyrosine	Rb	Poly	N9528	AB_260814	Sigma	1:8000
NRGN	Neurogranin	Rb	Mono	ab230154	NA	Abcam	1:4000
Olig2	Oligodendrocyte transcription factor 2	Rb	Poly	AB9610	AB_570666	MPS	1:4000
PCNA	Proliferating cell nuclear antigen	Ms	Mono	ab29	AB_303394	Abcam	1:250
PV	Parvalbumin	Ms	Mono	P3088	AB_477329	MPS	1:2500
Reelin	Reelin	Ms, CL142	Mono	MAB5366	AB_2285132	MPS	1:1000
S100	S100 calcium‐binding protein family	Rb	Mono	ab52642	AB_882426	Abcam	1:2000
SMI‐312	Phosphorylated neurofilament, pan axonal	Ms	Mono	837904	AB_2566782	BL	1:1000
SMI‐32	Non‐phosphorylated neurofilament 32	Ms	Mono	801701	AB_2564642	BL	1:1000
SNAP25	Synaptosomal‐associated protein, 25 kDa	Ms	Mono	836304	AB_2566521	BL	1:3000
SOM	Somatostatin	Sh	Poly	20C‐CR2056SP	AB_1288773	Fitz	1:3000
SOX2	SRY‐box transcription factor 2	Rb	Poly	AB5603	AB_2286686	Millipore	1:5 K
SOX5	SRY‐box transcription factor 5	Rb	Poly	AB94396	AB_10859923	Abcam	1:2000
SP	Substance P	Rt	Mono	sc‐21715	AB_628299	SC	1:250
SYP	Synaptophysin	Rb	Mono	ab32127	AB_2286949	Abcam	1:1000
TBR1	Brain‐specific T‐box TF1	Rb	Poly	ab31940	AB_2200219	Abcam	1:1000
TBR2	Brain‐specific T‐box TF2	Rb	Poly	ab23345	AB_778267	Abcam	1:500
TH	Tyrosine hydroxylase	Ms	Mono	P40101	AB_2617184	Pfz	1:1000
Vimentin	Vimentin	Rb	Mono	ab92547	AB_10562134	Abcam	1:5 K

*Note*: Research Resource Identifiers (RRID) are provided. Host species are abbreviated as follows: Rb for rabbit, Ms. for mouse, Rt for rat, and Sh for sheep. Antibody type is indicated as “Mono” for monoclonal, and “Poly” for polyclonal.

Abbreviations: BL, BioLegend; CST, cell signaling technology; DAKO, Agilent DAKO; Fitz, Fitzgerald; ImmS, ImmunoStar; MBS, MyBioSource; MPS, Millipore Sigma; NVS, NOVUS, Pfz, Pelfreeze; RDS, R&D systems; SC, Santa Cruz Biotechnology; Sigma, Sigma Aldrich; TF, ThermoFisher; Wako, Fujifilm Wako.

#### 2.2.1 Fixation, cutting, and storage

Processing of embryonic tissue has been, for the most part, very similar to that of adult tissue. However, adjustments are always necessary because of specimen size, the higher water content, and less neuropil of the embryonic tissue, which makes it more challenging to stain while preserving structural integrity, etc. General procedures are described below, but additional details can be found in Duque et al. (2025) and Mendoza‐Silva et al. (2026) or requested from the corresponding author.

Older embryos may be transcardially perfused with 4% paraformaldehyde (PF) in 0.1 M phosphate‐buffered saline (PBS). Otherwise, most embryonic brains are fixed by immersion in the same fixative. After fixation, tissue is cryoprotected in 20%–30% sucrose in 0.1 M PBS and subsequently frozen in isopentane and stored at −80°C. Later, brains are cut at 50 μm on a freezing microtome (Leica). So far, most brains have been cut in the coronal plane. When embryos are very young the whole head may be processed (Duque et al., 2025). Sequential sections are histologically stained with Nissl, reacted for acetylcholinesterase (AChE), or immunohistochemically treated. Processing is either done in‐house or the samples are sent to FD Neurotechnologies (Columbia, MD) for processing. Independent of location, all samples are processed nearly identically. If the samples are sent to FD, they are sent via overnight priority shipping, cold, wrapped in gauze (non‐woven 4″ × 4″ sponges), and sealed in a 250‐mL Nalgene jar filled with cold 20%–30% sucrose in PBS. The fetuses and postnatal animals for EM analysis (Collection 5) were, in general, transcardially perfused with a mixture of 4% PFA and 0.5%–1% glutaraldehyde in PBS, and brains were postfixed with 1% osmium tetroxide, dehydrated, and embedded in Epon‐Araldite epoxy resin.

#### 2.2.2 Nissl histochemistry

In general, sections are mounted on gelatin‐coated or Superfrost Plus (charged) glass slides and allowed to dry. Staining is done after the sections are defatted in a series of graded dilutions of ethanol. Because fat content is low in embryonic brains, treatment varies substantially according to age but is, on average, from 1 to 3 min. In some cases (very young embryos), this step can be omitted or reduced to a minimum. Sections are usually rehydrated in reverse order from the alcohol series to arrive again at double‐distilled water. Depending on how dark one may want to stain the sections, they are put into the Nissl solution between 1 and 10 min (compared to 5 and 30 min used in adult tissue). The Nissl solution commonly consists of 0.1 M sodium acetate (SA), 0.2 M formic acid (FS), and 0.5% aqueous cresyl violet (ACV) mixed in the following proportion: 30 mL + 150 mL + 300 mL of SA, FS, and ACV correspondingly. Sections are cleared in xylene and coverslipped with Permount® (Fisher Scientific). At FD, the sections are stained with FD cresyl violet solution™, and the results, as per our observation, are identical to what we obtain in‐house.

#### 2.2.3 AChE histochemistry

AChE histochemistry was performed similarly to that described for adult tissue (Duque et al., 2025; Mendoza‐Silva et al., 2026). In short, free‐floating sections are processed with the copper thiocholine method originally described by Hedreen et al. (1985), implemented in the NHP as described by Green and Mesulam (1988). After washes in 0.01 M PBS (pH 7.4), sections are pretreated in 0.05 M acetate buffer (pH 5.3) for 3 × 3 min and then incubated in solution A, containing SA, copper sulfate, glycine, ethopropazine, and acetylthiocholine for 12–24 h. After further rinsing and incubation in solution B (containing sodium sulfide, pH 7.8), followed by incubation in solution C (containing silver nitrate), the sections are re‐fixed overnight in 4% PFA in 0.1 M PBS (pH 7.4). Finally, the sections are mounted on gelatin‐coated or Superfrost Plus slides, dehydrated, cleared in xylene, and coverslipped with Permount® (Fisher Scientific).

#### 2.2.4 Periodic acid–Schiff (PAS)–Alcian histochemistry

PAS–Alcian Blue histochemistry excels at staining extracellular matrix (ECM) by staining for acid and neutral mucopolysaccharides/glycosaminoglycans. It is therefore very useful in labeling the ECM‐rich subplate (SP), and hence, it is a transient staining that disappears later in development (Junaković et al., 2023; Kostović et al., 2014; Kostović & Judas, 2002). In general, sections are cleaned in an ascending and then descending series of alcohols, followed by staining in Alcian Blue solution at pH 2.5. This is usually done between 20 and 30 min. After rinsing, the sections are treated (oxidized) in 0.5% periodic acid for ~5 min. After further rinsing, the sections are reacted with Schiff solution for 15–30 min in the dark, then rinsed, and counterstained with Hematoxylin for 1–5 min. After a final rinse, the sections are dehydrated, cleared in xylenes, and coverslipped with Permount® (Fisher Scientific). Depending on embryonic age and the consequent significant differences in water and neuropil content, adjustments to incubation times and strengths of the different reagents may be necessary.

#### 2.2.5 Immunohistochemistry

For further details, see our companion paper by Mendoza‐Silva et al. (2026). In short, we try to do all rinses and incubations on free‐floating sections in 0.1 M PBS at room temperature (RT), but with very young tissue, this is not always possible, and the processing must be done on glass‐mounted sections (Duque et al., 2025). Stored and cryo‐protected sections are rinsed to remove the cryoprotectant before 0.1%–0.5% hydrogen peroxidase treatment (5–10 min, or less) to inactivate endogenous peroxides. A parallel series of sections spaced between 0.4 and 1 mm apart is processed for the same antibody. The sampling rate depends on brain size, which in turn depends on age. Sections are incubated for ~48 h in a solution containing the primary antibody (Table 2) and with 0.05%–0.2% Triton‐X. After rinsing, incubation in the biotinylated secondary antibody is usually for 4–24 h. Dilutions vary but are on average 1:200–500 and may contain 1%–5% serum from the animal host of the secondary antibody (or bovine serum albumin). Final incubation is in avidin–biotin–peroxidase complex for 4 h (Vectastain elite ABC kit, Vector Labs, Burlingame, CA, USA). Labeling is visualized by 0.05% 3′,3′‐diaminobenzidine (DAB) as a chromogen, precipitated by 0.01% hydrogen peroxide. The DAB reaction protocol varies little from specimen to specimen or stain to stain and, in more general terms, is performed according to the avidin‐biotin complex protocol provided by Vector Labs (Burlingame, CA) and/or the method of (Hsu & Raine, 1981) with the Vectastain elite ABC Peroxidase kit. After thorough washes, the sections are mounted on glass slides, dehydrated in ethanol, cleared in xylene, and coverslipped with Permount® (Fisher Scientific). DAB immunoreacted sections have the advantage that they can be stored at RT. The data in Table 2 are the abbreviated and summarized most current general information. If further or more precise information is needed, please contact the corresponding author.

**TABLE 2:** Summary of embryonic specimens already available.

Brain no.	B163	B111	B121	B177	B117	B165	B116	B175	B112	B174	B109^a^	B169	B110^a^	B120	B166	B180^b^	B118^c^	B173^c^	B78	B114^c^	B107^b^
					B108^a^																
Sex	F	NA	F	F	M‐NA^a^	F	M	F	M	F	NA^a^	F	NA^a^	F	F	M	F	M	M	F	F
Stain/Age	E42	E49	E57	E62	E70	E79	E90	E93	E95	E96	E97	E102	E105	E110	E120	E130	E131	E140	E143	E149	E150
5‐HT				X				X							X	X	X	X		X	X
AChE	X	X	X		X	X	X	X	X	X	X	X	X	X	X	X	X	X			
BIII‐tubulin									X		X	X	X	X	X	X	X	X			
BrdU					X						X		X								
CB				X				X							X	X	X	X	X	X	X
CCK																		X	X		X
ChAT				X						X				X	X		X	X	X	X	X
CR				X											X		X	X	X	X	X
DCX										X		X						X			
Fibronectin	X	X	X		X	X	X		X		X	X	X	X	X	X	X	X			
GFAP	X	X	X		X	X	X		X	X	X	X	X	X	X	X	X	X			
Iba1								X							X	X	X	X	X	X	X
Ki67	X	X	X		X	X	X		X	X	X	X		X	X	X	X	X			
MBP					X						X		X		X	X	X	X	X	X	X
NCAN					X						X		X								
NeuN				X				X		X				X	X	X	X	X	X	X	X
Nissl	X	X	X	X	X	X	X	X	X	X	X	X	X	X	X	X	X	X	X	X	X
NOS				X				X							X	X	X	X		X	X
NPY				X				X		X				X	X	X	X	X	X	X	X
NRGN															X	X	X	X	X	X	
Olig2								X							X	X	X	X	X	X	X
Pas–Alcian	X	X	X		X	X	X		X		X	X	X	X	X	X	X	X			
PCNA										X					X			X			
PV				X				X							X	X	X	X	X	X	X
Reelin									X			X	X	X	X	X	X	X			
S100																			X		
SMI‐312	X	X	X	X	X	X	X	X	X	X	X	X	X	X	X	X	X	X	X		
SMI‐32				X				X		X		X			X	X	X	X	X	X	X
SNAP25	X	X	X		X	X	X		X		X	X	X	X	X	X	X	X			
SOM															X	X	X	X	X	X	X
SOX2	X	X	X		X	X	X		X	X	X	X	X	X	X	X	X	X			
SOX5									X			X	X	X	X	X	X	X			
SP				X											X	X	X	X		X	X
SYP	X	X	X		X	X	X		X		X		X	X	X	X	X				
TBR1	X	X	X		X	X	X		X	X	X	X	X	X	X	X	X	X			
TBR2	X	X	X		X	X	X		X	X	X	X	X	X	X	X	X	X			
TH				X				X							X	X	X	X	X	X	X
Vimentin										X		X			X			X			
No. of series	12	12	12	13	12	12	12	15	15	15	16	17	17	18	34	30	31	34	15	17	18
					15																
No. of sections	132	144	240	377	432	480	432	690	735	660	160^a^	731	170^a^	774	1632	NA	1643	1904	1035	935	NA
					150^a^																

*Note*: Total number of images, including the ones currently being scanned: 13,456. Abbreviations for the different stains (*n* = 38) appear in alphabetical order in the left column, and the corresponding embryo ages appear in subsequent columns, left to right, in ascending order. All embryos were obtained from the Rakic rhesus macaque breeding colony at Yale, and embryonic ages are accurate at ±1.5 days (*n* = 22; does not include *n* = 2 (1 M, 1 F) specimens being prepared for histology). Table data are the current data as of July 2025.

^a^ Not complete rostro‐caudal series, sections from selected brain blocks only, available upon request.

^b^ Immunohistology in process, and hence the exact number of sections is not yet available (NA).

^c^ Being scanned.

During optimization, different antibodies for the same marker but manufactured by different companies, or with different primary hosts, and/or at different dilutions, may have been tried. The antibodies and dilutions that we empirically determined to be best, and therefore, the ones we continue to use, are listed in Table 1. Further details can be requested from the corresponding author.

As reported in our companion paper (Mendoza‐Silva et al., 2026), there are many caveats, including that manufacturers or vendors discontinue products, change distributors, make changes that affect the catalog number of a product, etc. In addition, the processing of embryonic tissue, although in general similar to that of adult tissue, can be much more difficult and capricious, as naturally early in development the expression of many of the markers we use is rapidly changing and procedural adjustments may be needed.

### 2.3 Digitization, digital data storage, and public web galleries

For further details, see Duque et al. (2025). In short, for digitization, the MBRC uses primarily an Aperio CS2 high resolution scanner (Leica) and the images are acquired at 20× magnification using an Olympus UPlanSApo 20×/0.75 NA objective lens infinity corrected—wide field (∞/0.17 FN26.5), which provides a resolution of ~0.50 μm/pixel, at a working distance of 0.65 mm. 40× can be achieved through a 2× optical changer. The digital images are formatted as Aperio ScanScope Virtual Slides (SVS) and are Big‐Tagged image file format (TIFF) files. The SVS images include image data stored using lossy JPEG2000 compression and a macro view in low resolution stored with lossless Lempel–Ziv–Welch (LZW) compression. The scanner sends the images to our storage repository using the ImageID value displayed within the eSlideManager web interface. Later, we convert the SVS image files to Deep Zoom image format using libvips, an open‐source image processing library compatible with OpenSlide image files. The OpenSlide enables libvips to operate as a bridge between Aperio and Deep Zoom and/or other International Image Interoperability Framework (IIIF) image formats. Our digital output (the public galleries viewable in Collection 6) is Deep Zoom images in a custom‐built, responsive, labeled image frame template.

### 2.4 Tritiated thymidine ( ^3^H‐TdR) cases in collection 1

More than *n* = 100 cases from tritiated thymidine (^3^H‐TdR) injections in pregnant NHP females are contained in the MBRC Collection 1. Of these cases, we have so far corroborated viability for research in *n* = 99 cases. These include specimens that vary in injection time from early (~25 days) to late (~140 days) in gestation (*n* = 61 total) in which embryos were collected by C‐section at different intervals post injection distributed as follows: ~1 h (*n* = 19), 3 days (*n* = 12), 7 days (*n* = 17), and 14 days (*n* = 13). Also, injected within the same period but with postnatal brains collected at various ages, including adult, are *n* = 21 cases. In addition, Collection 1 contains *n* = 17 cases in which ^3^H‐TdR injections were given directly to the postnatal animal with brain harvesting at various survival periods, including years later. Viability of the sections remains to be determined in approximately another *n* = 35 cases that require extensive refurbishing, usually replacement of the glass coverslip and, in some cases, renewed Nissl counterstaining.

### 2.5 EM blocks in collection 5

Collection 5 contains *n* > 100 cases in which blocks of tissue from various cortical and subcortical regions, the cerebellum, brainstem, retina, and other regions were prepared for EM. Collection 5 contains samples from most of the original specimens in Collection 1, but in addition contains EM blocks from other experiments, some in other Collections of the MBRC. Overall, so far, we have classified and organized >3000 EM blocks, and we have identified the normative data of the specimen from which they originate. Of the total number of samples, we have blocks of proven quality for ~*n* = 50 embryonic cases, and many more remain to be tested.

### 2.6 Human fetal brain tissue processing

Human fetal brain tissue ranging from 17 to 26 post‐conceptional weeks (PCW) is part of the Zagreb Neuroembryological Collection, with all procedures approved by relevant IRBs. Brains were immersion‐fixed in 4% paraformaldehyde, embedded in paraffin, and sectioned for histological analysis using standard Cresyl‐violet (Nissl) staining to define cytoarchitectonic boundaries, PAS–Alcian blue for ECM localization (described in Duque et al., 2016; Kostović & Judas, 2002), and TBR1 immunohistochemistry (Junaković et al., 2023). All tissue sections were scanned and analyzed using a high‐resolution NanoZoomer 2.0RS digital slide scanner.

## 3 RESULTS

First, a list of stains currently available in the different samples is provided in Table 2 to inform the reader as to the volume and scope of the embryonic materials being prepared. Then, through examples, we illustrate how embryonic materials in these datasets are actively being used in research.

**FIGURE 2:**
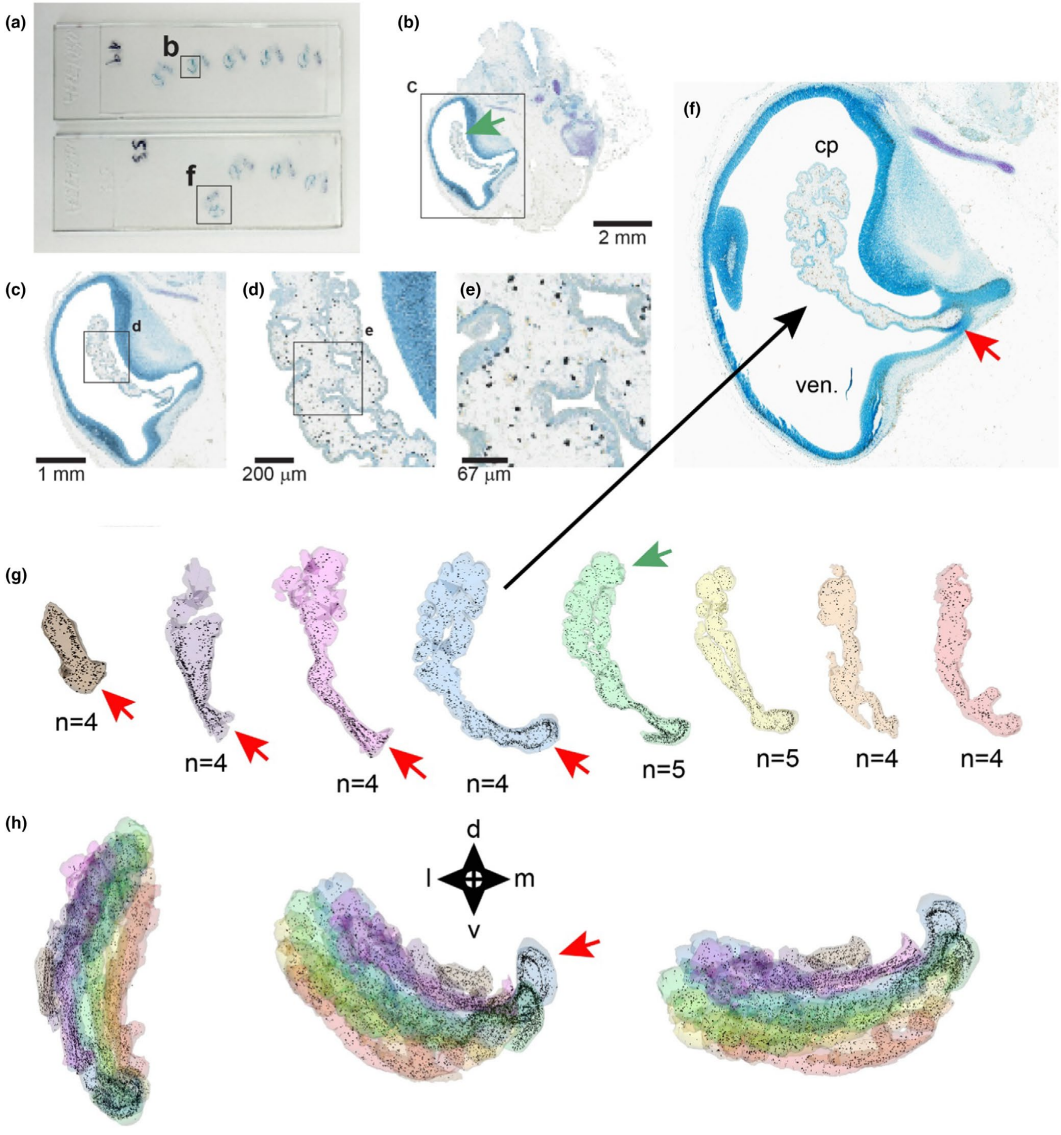
3D Spatiotemporal mapping of choroid plexus (cp) development in relationship to cortex. (a) Example of 2–9 glass slides containing 34 brain sections cut at ~30 μm thick. These are from an embryo, obtained by C‐section, 1 h after the pregnant F was injected with ^3^H‐TdR at E41. (b–f) Sections developed by autoradiography show positive cells in the cp (black dots), indicative of cell birth at the time of injection. (g, h) 3D computational reconstructions of the cp using the program “Reconstruct.” The red arrows point to the CP attachment. The green arrow points to cp in a single section in (c) and the reconstructed set in (g). For orientation, the compass rose shows d, dorsal; v, ventral; l, lateral; m, medial. Cerebrospinal fluid (CSF) production by the CP plays a crucial role in cortical development. We are investigating this relationship. Materials from Collection 1, case 060673A; that is, the embryo was obtained on June 6, 1973; a fine example of the usefulness of archived materials to conduct new research. Panels (a–f) are from figure 5 in Duque et al. (2025).

Figure 2 illustrates a study of choroid plexus (CP) development in relation to cortical development and the production of cerebrospinal fluid (CSF). This is using Collection 1 ^3^H‐TdR materials developed by autoradiography decades ago. This study within the MBRC group is specifically testing our capacity to use artificial intelligence (AI) powered analysis to investigate ependyma cell development using ^3^H‐TdR materials. Because the CP consists of an epithelial lining and internal stroma that undergo major structural changes during embryonic and early postnatal development, the quantitative analysis of these changes is challenging in archived tissue due to variable preservation, sectioning, and staining quality. For this investigation, we are using a hierarchical detection model and a blanket detection model with regional segmentation. Our results so far indicate the hierarchical model produces more accurate results, reducing stroma false positives and matching manual assessments. Our algorithm is being used for 3D reconstruction of the CP location in relation to cortical coordinates (Burke et al., 2025).

**FIGURE 3:**
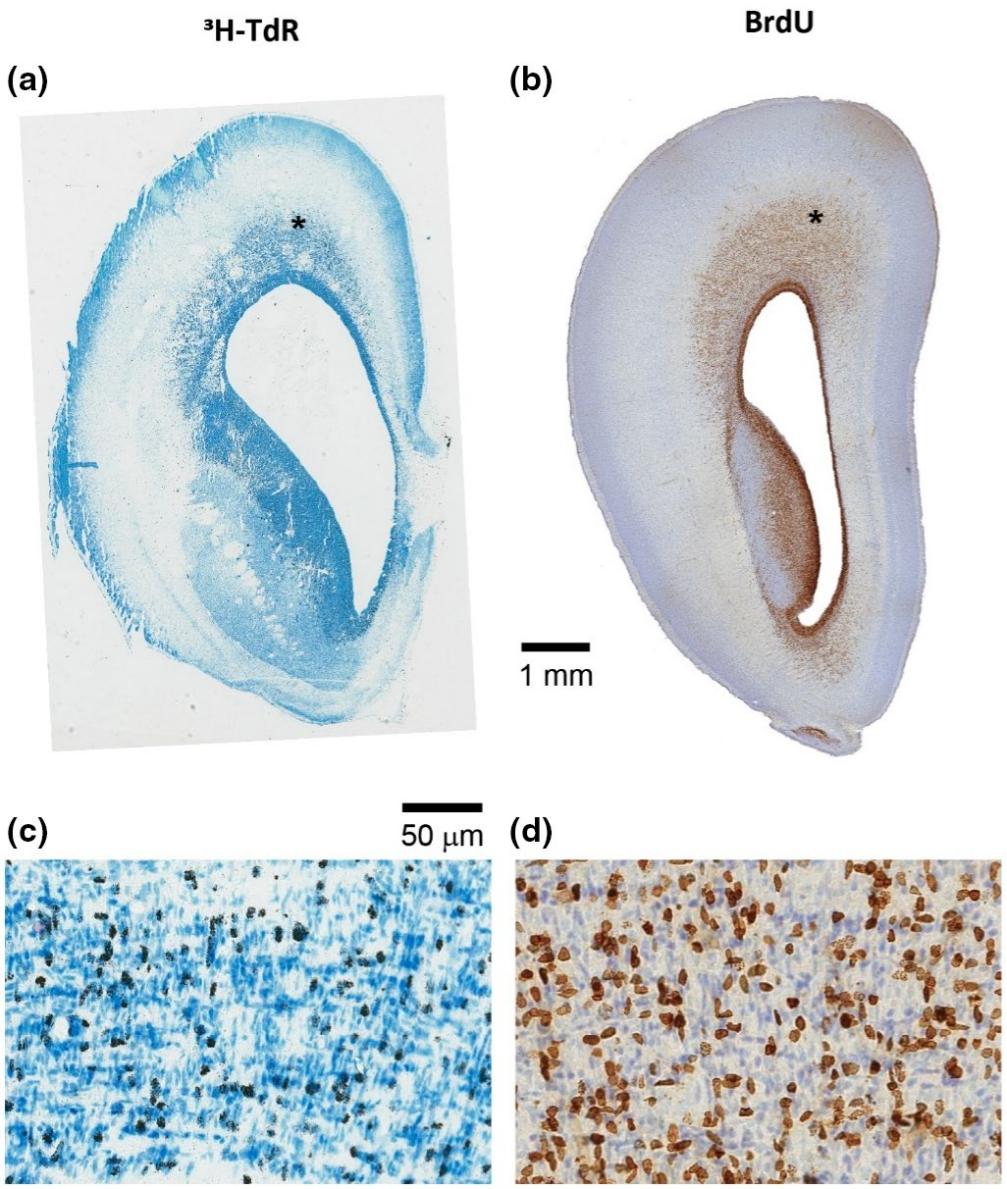
Comparison of staining (a, c) ^3^H‐TdR versus (b, d) BrdU in the embryonic cortical wall 1 h after injection at E69 and E70, respectively. Although these frontal sections are at slightly different rostro‐caudal levels, this figure illustrates our capacity to analyze, compare, contrast, and exploit results obtained from materials in Collection 1 and those being processed now and incorporated or to be incorporated into Collection 6. ^3^H‐TdR is from case 022070D, developed by autoradiography in February 1970; BrdU is from a case developed by immunohistochemistry in May 2019. This example of the combined use of older and newer archived materials processed by different methods serves to illustrate that caution is necessary in the interpretation of results because the methods, although globally in agreement, still show differences in the numbers and distributions of labeled cells (higher in BrdU).

Figure 3 illustrates our capacity to compare results obtained from materials in collection 1 and those more recently obtained using BrdU. Comparison of results between ^3^H‐TdR and BrdU not only highlights important methodological considerations (Duque & Rakic, 2015) but has proven useful in the proper interpretation of the birth, migration, and distribution of neurons to different cortical areas. As we are preparing to add a significant amount of BrdU‐labeled materials to Collection 6, Figure 3 demonstrates that the birth dating approaches show globally similar labeling patterns, but that there are differences that need to be considered when analyzing cell numbers and distributions. Here, notice the higher number of labeled cells in BrdU (not affected by the slight difference in the rostrocaudal level). Our investigation comparing the two methods in the prefrontal, limbic, primary motor, primary somatosensory, and visual cortex has already provided ample quantitative evidence that the methodology used has implications for the interpretation of results in terms of neuronal numbers, their migration, placement, subsequent connectivity, function, and survival (Duque & Rakic, 2011).

**FIGURE 4:**
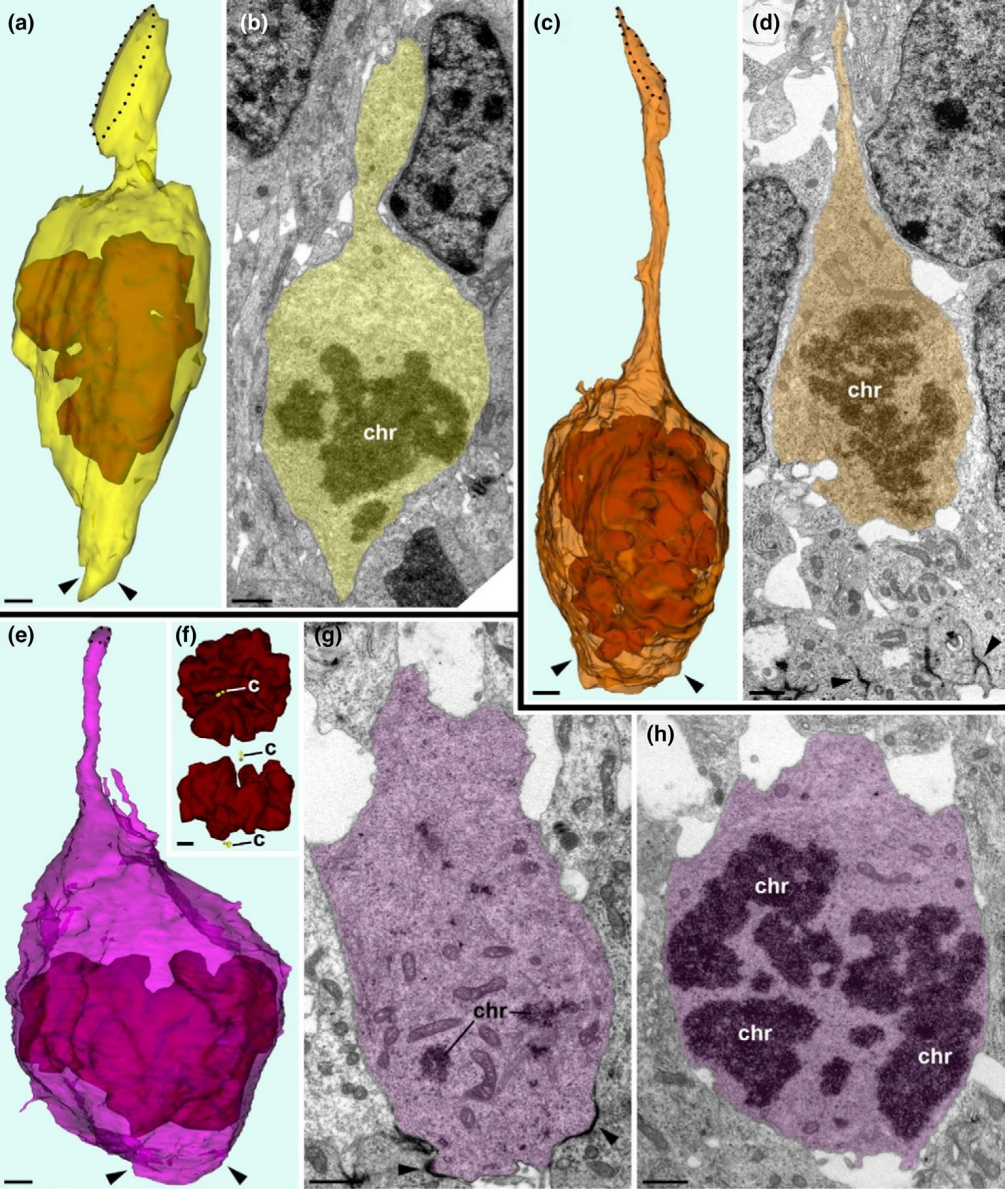
Electron microscopy 3‐D reconstruction from serial ultrathin sections of three mitotic cells in the neocortical VZ of a 55‐day‐old rhesus monkey embryo. Two of the cells are in the metaphase, as evident by condensed chromosomes (chr) (a–d), and the third is in anaphase (e–h), evident with the disk of chromosomes in the middle of the cell body and opposing centrioles (c) that are shown in two reciprocally perpendicular projections in (f). Notice that all these cells emit incompletely reconstructed radial processes (last profiles of the truncated processes are indicated with black dotted lines). The reconstructed cells and characteristic electron micrographs of them are highlighted with corresponding semitransparent colors (yellow, orange, and magenta). Conglomerates of chromosomes (chr) are shown inside the cell bodies in the 3‐D images. The ventricular surface is identifiable with the row of adherens junctions that are indicated with arrowheads in the micrographs and the 3‐D reconstructions. Scale bars, 1 μm. Collection 5.

Figure 4 illustrates the EM 3‐D reconstruction of three mitotic cells in the neocortical VZ of a 55‐day‐old rhesus monkey embryo from serial ultrathin sections obtained from archived materials in Collection 5. The study, still inconclusive, is looking into differences in the establishment of polarity between symmetric and asymmetric cell division. An additional reason for this example is to illustrate the high quality and viability of the materials archived in Collection 5. EM blocks embedded in Epon‐Araldite epoxy resin, in theory, can last forever, like prehistoric insects in amber fossilized tree resin; it seals the samples, protecting them from degradation.

**FIGURE 5:**
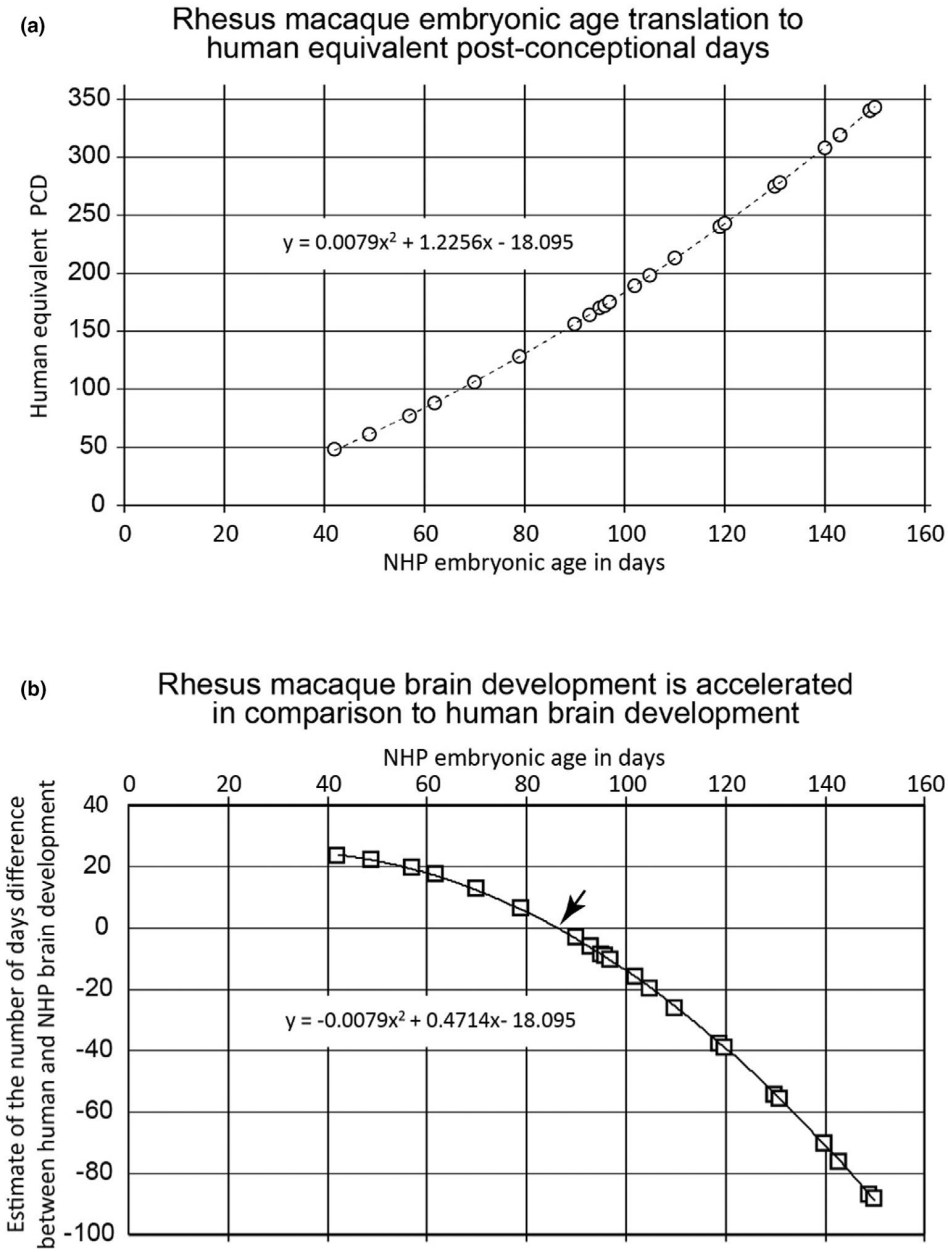
Comparison of the embryonic brain developmental age between humans and NHP. (a) Human post‐conceptional days (PCD) age equivalent for each of the *n* = 24 NHP embryos in this report (*n* = 22 different ages with 2 repetitions at E70 and E140, as shown in Figure 1). (b) The equivalent developmental time was calculated using the “Translating time across developing mammalian brains” application available at https://www.translatingtime.org/translate/ (Workman et al., 2013). See text for further details.

Figure 5 illustrates a developmental age comparison between human and rhesus macaque using plots constructed from data provided by Workman et al. (2013), which is available in the “Translating time across developing mammalian brains” application accessible at https://www.translatingtime.org/translate/. These plots provide an important temporal basis for comparison. The developmental age differences between the rhesus macaque and human, within the embryonic ages currently being processed in the MBRC, are provided in Table 3. Figure 5a indicates that a polynomial of order 2 provides the best match to relate rhesus macaque embryonic age to its human equivalent, within the age range available in the current sample. Figure 5b shows the heterochrony between human and rhesus macaque brain development by plotting the number of days difference between corresponding gestational ages. This illustrates the same data as shown in Figure 5a but highlights an acceleration in NHP neurodevelopment with respect to human starting at approximately 86 days in gestation (marked with an arrow). Of course, this is known a priori to be true given the observed level of development at time of birth and that the average gestation is 165 days in rhesus macaque and 280 days in human. Notice that, numerically, only the coefficient of the first power of *x* changes, but arithmetically, the first and third terms change sign.

**TABLE 3:** Post‐conceptional days.

Rhesus	Human
42	48
49	61
57	77
62	88
70	106
79	128
90	156
93	164
95	170
96	172
97	175
102	189
105	198
110	213
119	240
120	243
130	275
131	278
140	308
143	319
149	340
150	343

**FIGURE 6:**
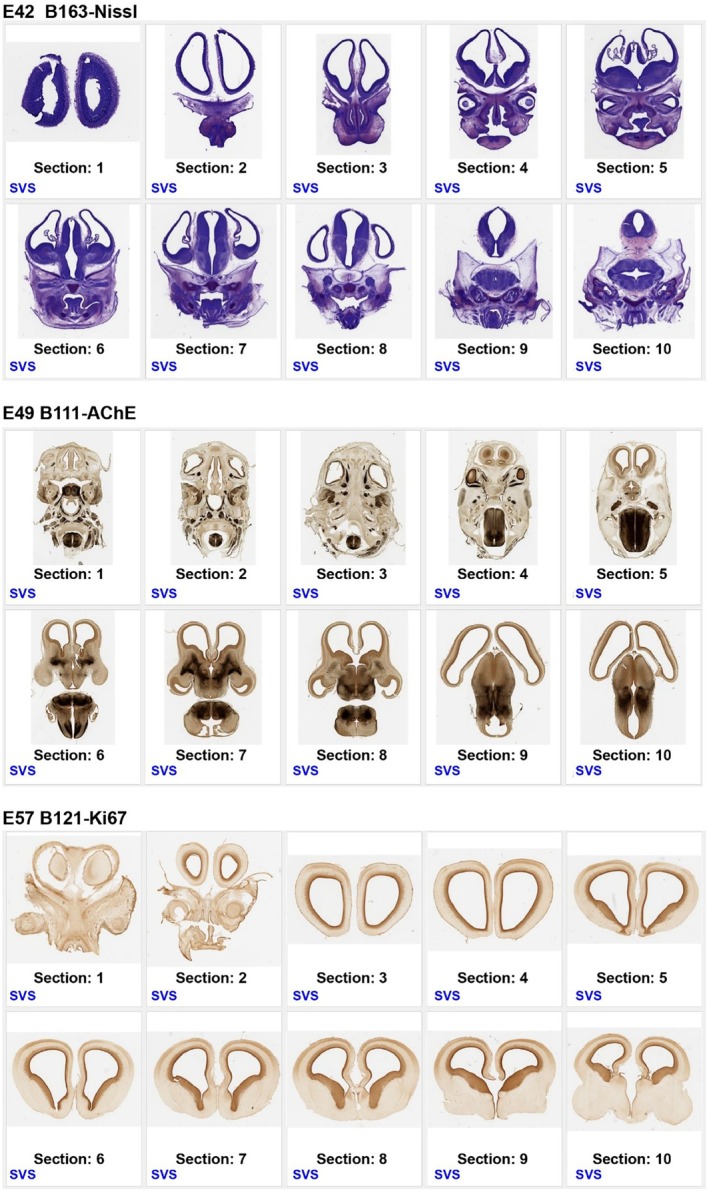
Examples of the sequential histological images of rostral to caudal coronal sections of three different stains at three different embryonic ages. These galleries are already incorporated into Collection 6 of the MBRC (https://macbraingallery.yale.edu/collection6/). Each image can be toggled to a full page and viewed at high magnification. Images can be downloaded in different formats. At the lower left of each image is an “svs” link that allows them to be opened in the original acquisition format and explored with the free Leica software ImageScope.

Figure 6 provides a macro‐view of some of the galleries prepared for incorporation in Collection 6 of the MBRC. The galleries containing embryonic tissue are being prepared in the same manner as those that contain adult tissue, illustrated in our companion paper (see: https://macbraingallery.yale.edu/collection6/). Each image is zoomable and downloadable. In Collection 6, there are already *n* = 14 embryonic specimens and correspondingly >10,000 images that are publicly available (also see Table 2).

**FIGURE 7:**
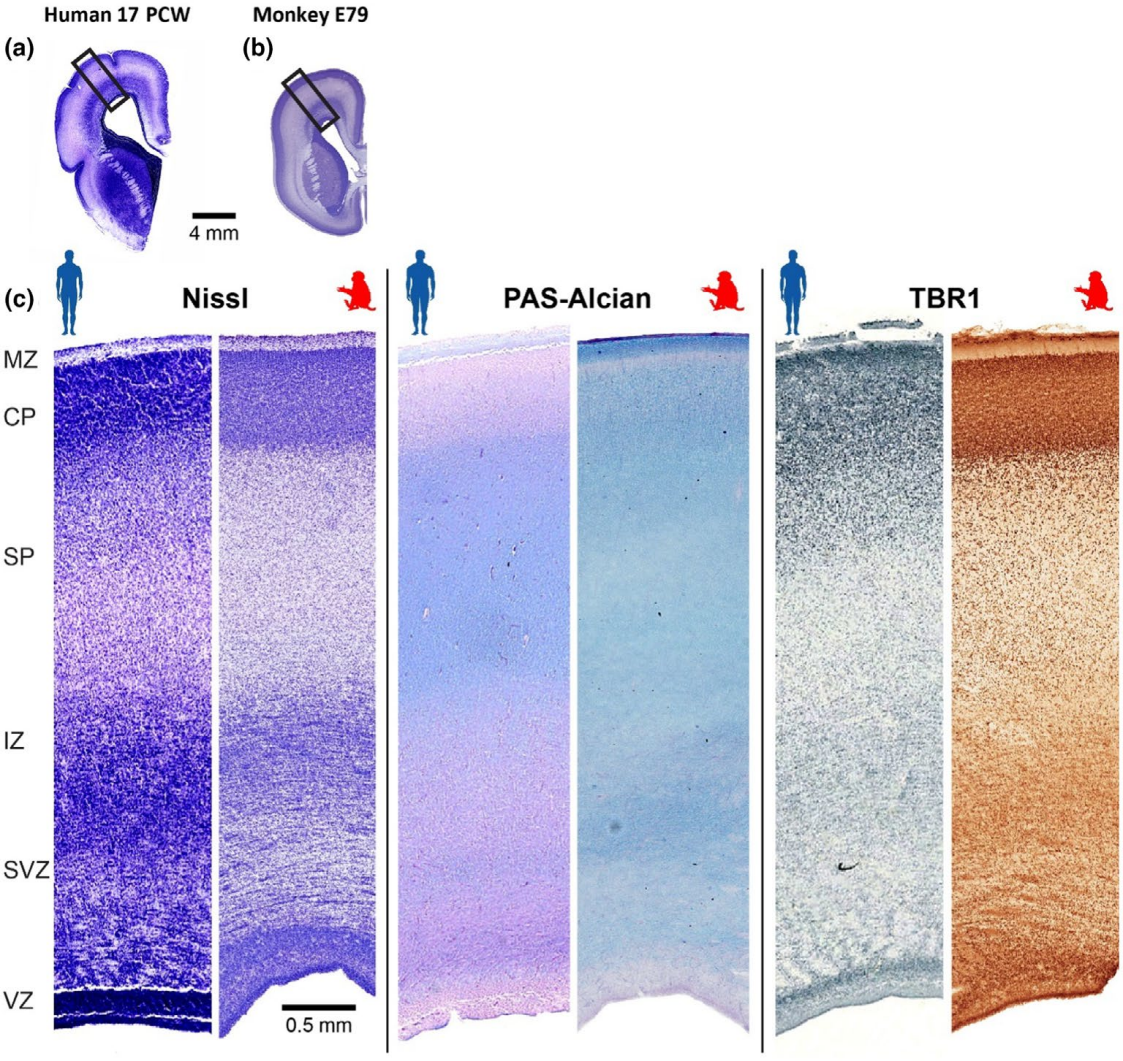
Earlier mid‐gestation‐cytoarchitectonic and laminar pattern comparison in the dorsolateral region of the developing frontal cortex in (a) humans (17 PCW) and (b) Macaca mulatta (E79). (c) Nissl and PAS–Alcian staining highlight the prominent SP zone, rich in ECM, while TBR1 expression serves as a marker of deep‐layer projection neurons, now particularly rich in layer VI and the SP. Notice that the patterns are similar in both species. Scale in between (a) and (b) applies to both panels. Scale bar in monkey Nissl panel applies to human Nissl and all PAS‐Alcian and TBR1 panels. CP, cortical plate; ECM, extracellular matrix; IZ, intermediate zone; MZ, marginal zone; PAS, periodic acid–Schiff; SP, subplate; SVZ, subventricular zone; VZ, ventricular zone.

**FIGURE 8:**
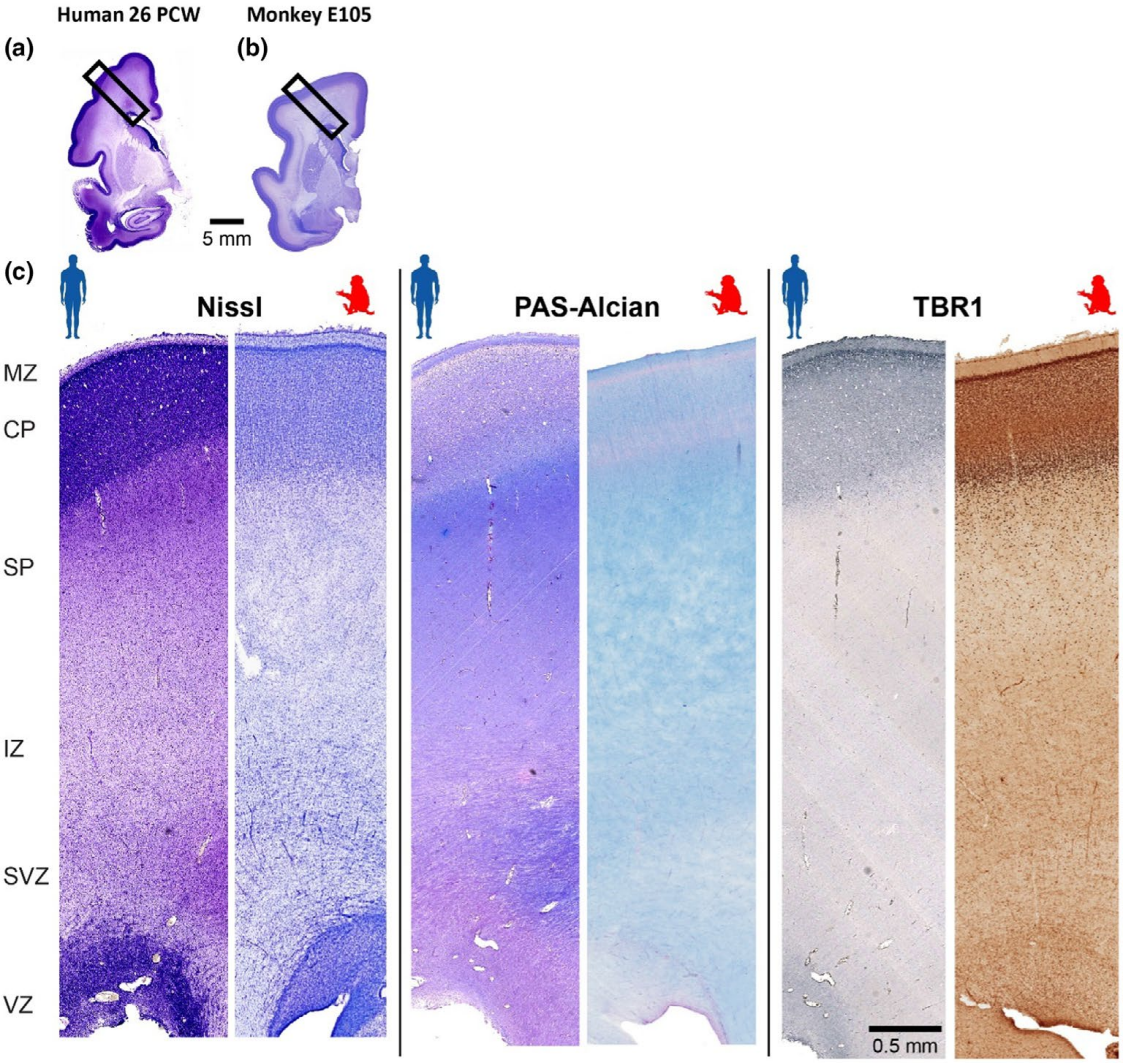
Later mid‐gestation cytoarchitectonic and laminar pattern comparison in the dorsolateral region of the developing frontal cortex in (a) human (26 PCW) and (b) *Macaca mulatta* (E105). Folding of the cortex and the thickness of the CP are now more prominent than in the earlier example. (c) Nissl, PAS–Alcian, and TBR1 staining. Nissl staining illustrates the cytoarchitectonic organization of transient fetal cortical layers. PAS–Alcian staining still highlights the prominent zone rich in ECM. Expression of TBR1, a marker of future cortical layer VI, is still seen in the SP but also more clearly than before in the deeper CP. CP, cortical plate; ECM, extracellular matrix; IZ, intermediate zone; MZ, marginal zone; PAS, periodic acid–SchiffSP, subplate; SVZ, subventricular zone; VZ, ventricular zone. Scale bar between (a) and (b) applies to both panels. Scale bar in human TBR1 applies to monkey TBR1 and all Nissl and PAS–Alcian panels.

Figures 7 and 8 provide a comparison of the cytoarchitecture and laminar patterns at early and later mid‐gestation of the dorso‐lateral frontal cortex between human and rhesus macaque. The corresponding developmental ages are matched as close as possible within the limited amount of materials available. Materials using Nissl, PAS‐Alcian, and TBR1 highlight the importance of having a rich repertoire of stains for interspecies comparisons at different developmental ages, which allow the mapping of normal cortical changes that occur during these transformative embryonic periods. These examples illustrate collaborative ongoing studies between the MBRC and our colleagues associated with the human brain collection at the University of Zagreb.

## 4 DISCUSSION

Progress in the study of NHP cerebral cortex development has been constrained by the limited availability of complete and organized histological developmental datasets (Duque et al., 2025; Shinmyo et al., 2022). This has been an important driving force for the addition of embryonic brains to the MBRC Collection 6. As the Collection grows, additional embryonic brains will be added to ensure sufficient information on developmental stages, marker diversity, and sex balance. Also, the established datasets guarantee accessibility in the long term (Duque et al., 2025; Mendoza‐Silva et al., 2026), hence reducing the gaps that restrict study reproducibility and the establishment of developmental standards for both basic and translational neuroscience.

Current research into NHP development covers a diverse range of research questions from cellular architecture to longitudinal neuroimaging. Practical issues and lower cost have lately been driving forces in favor of the marmoset and hence, in the common marmoset, different features of early corticogenesis have already been identified, including inner and outer subventricular zones, persistent postnatal cortical progenitors, delayed V1 development and early thalamocortical innervation of the subplate, which, as in other species, serves as a dynamic waiting compartment for ingrowing afferent systems during early circuit assembly (Hoerder‐Suabedissen & Molnár, 2015; Homman‐Ludiye & Bourne, 2020). These cellular insights are complemented by genetic expression resources, such as profiling systems that analyze the neonatal marmoset brain (Kita et al., 2021). Comparative studies between species (marmosets, macaques, mice, and humans) further indicate that although many cell types in the primary motor cortex are shared among mammals, gene expression and abundance differ considerably (Bakken et al., 2021). Parallel efforts in the rhesus macaque have focused on neuronal maturation in the developing prefrontal cortex (Gao et al., 2025). This work is also supported by neuroimaging research that provides reference datasets for fetal development. For instance, MRI and label maps at E85, E110, and E135 have been used to analyze growth and white matter maturation in the macaque (Liu et al., 2020). Similar longitudinal MRI studies have captured the second half of macaque pregnancy (Karpf et al., 2025). Beyond the macaque, MRI atlases for the marmoset enable the tracking of brain growth from gestational week 8 through birth (Hikishima et al., 2013). Recent advances have also explored the creation of live ultrasound imaging of marmoset brain and heart development, which allowed the validation of a unique prolonged early stage that could be valuable as a primate reference for human counterparts (Soman et al., 2024). In addition, the NHP developmental genotype‐tissue expression (NHP‐dGTEx) NIH initiative is already establishing a database and tissue bank to study gene expression patterns that include the brain. This multi‐institutional effort seeks to establish a reference for gene expression in rhesus macaques and marmosets for comparison to healthy developmental, neonatal, pediatric, and adolescent humans. They do not aim to provide immunohistology or protein expression as in the MBRC Collection 6.

Among the closest embryonic datasets to the ones currently being prepared in the MBRC are those of the Allen Institute for Brain Science. These currently incorporate 6 embryonic ages: E40, E50, E70, E80, E90, and E120 that have MRI imaging. Sections in the E40 to E90 were stained for Nissl, Ectoderm‐Neural Cortex 1 protein (ENC1), and growth‐associated protein 43 (Gap43). Sections at E120 were stained for Nissl and AChE. Each embryonic age contains 2 M and 2 F. The materials can be accessed via different links, including through the Blueprint NHP Atlas. In summary, their data encompass *n* = 24 specimens and a total of 5 stains. MacBrain has the same number of embryos already committed (see Tables 1 and 2 for details). Currently, Collection 6 provides access to *n* = 14 embryo brains encompassing 38 different stains and >10,000 images (https://macbraingallery.yale.edu/collection6/), all of them zoomable and downloadable; images from *n* = 3 partial sets (not rostral to caudal complete series) are available upon request, sections from *n* = 3 brains are being digitized and *n* = 2 brains are getting ready for histology. Every series includes specimen identifiers: age, sex, and stain. Web access promotes transparency and fosters the use and reuse of the materials, thereby positioning the MBRC as a reference for comparative research. As a reference point in humans, the DHARANI atlas provides a second‐trimester developmental reference that incorporates histological sampling within an interactive portal and provides multimodal imaging data for comparison of large developmental datasets, and utilization as a resource for data organization and annotation (Verma et al., 2025). Together, all these datasets promise a rich repertoire of embryonic ages and histological processing to assist in a host of NHP developmental studies essential to advance the field of neurodevelopment. Currently, the MBRC is providing the raw image data sets to the public but is not annotating brain regions, which, during development, change rapidly and dramatically. Annotation and 3D reconstructions using MBRC embryonic materials are not, per se, goals of the MBRC, although, privately, the materials are being used in this way by some investigators.

MBRC Collections 1 and 5 continue to be very unique, and we are not aware of similar publicly available datasets with the variety and number of samples these Collections afford. Of the >100 cases from tritiated thymidine (^3^H‐TdR) injections in pregnant NHP females contained in MBRC Collection 1, we have so far checked the quality of *n* = 99 cases; of these *n* = 61 cases encompass early (~30 days in gestation) to late injections (~135 days in gestation) in which the embryos were collected by C‐section at roughly 1 h (*n* = 19 cases), 3 days (*n* = 12 cases), 7 days (*n* = 17 cases), and 14 days (*n* = 13 cases) post injection. The Collection also includes ^3^H‐TdR injections from ~E25 to E140 with postnatal offspring brain harvesting (*n* = 21 cases) and postnatal ^3^H‐TdR injections with postnatal brain harvesting; the last 2 sets at various survival periods (*n* = 17 cases). The case is similar for the EM blocks available in Collection 5, of which >60 cases already inventoried and catalogued are embryonic.

Taken together, the different studies and different datasets show the utility of and need for NHP models to distinguish and establish primate developmental features that are species‐specific specializations often lost in other species. The study of brain development in rhesus macaque and in marmosets offers unique features that cannot be tracked in rodents, and altogether will hopefully help bridge the gap in our understanding of the complexities of human brain development and function.

## AUTHOR CONTRIBUTIONS

AD, ZK, and PR: Concept, design, and financial support. VMS, EB, LG, MR, JK, YMM, AD, and KMI: Data acquisition. AD, VMS, COM, JK, ZK, PR: Data analysis and interpretation. AD and VMS: Wrote the paper. PB: Developed, managed, maintained, and supported by MBRC technology under the direction of AD. AD is the director of the MBRC. All authors provided critical editorial revisions of the manuscript and approved the article.

## CONFLICT OF INTEREST STATEMENT

The authors declare no conflict of interest.

## Data Availability

The data that support the findings of this study are openly available in MacBrain Resource Center at https://macbraingallery.yale.edu/collection6/.
